# Secure and Reliable Big-Data-Based Decision Making Using Quantum Approach in IIoT Systems

**DOI:** 10.3390/s23104852

**Published:** 2023-05-18

**Authors:** Abir EL Azzaoui, Mikail Mohammed Salim, Jong Hyuk Park

**Affiliations:** Department of Computer Science and Engineering, Seoul National University of Science and Technology, Seoul 01811, Republic of Korea; abir.el@seoultech.ac.kr (A.E.A.); mikail@seoultech.ac.kr (M.M.S.)

**Keywords:** smart factory, IIoT, quantum algorithms, quantum neural network, quantum approximate optimization algorithm

## Abstract

Nowadays, the industrial Internet of things (IIoT) and smart factories are relying on intelligence and big data analytics for large-scale decision making. Yet, this method is facing critical challenges regarding computation and data processing due to the complexity and heterogeneous nature of big data. Smart factory systems rely primarily on the analysis results to optimize production, predict future market directions, prevent and manage risks, and so on. However, deploying the existing classical solutions such as machine learning, cloud, and AI is not effective anymore. Smart factory systems and industries need novel solutions to sustain their development. On the other hand, with the fast development of quantum information systems (QISs), multiple sectors are studying the opportunities and challenges of implementing quantum-based solutions for a more efficient and exponentially faster processing time. To this end, in this paper, we discuss the implementation of quantum solutions for reliable and sustainable IIoT-based smart factory development. We depict various applications where quantum algorithms could improve the scalability and productivity of IIoT systems. Moreover, we design a universal system model where smart factories would not need to acquire quantum computers to run quantum algorithms based on their needs; instead, they can use quantum cloud servers and quantum terminals implemented at the edge layer to help them run the desired quantum algorithms without the need of an expert. To prove the feasibility of our model, we implement two real-world case studies and evaluate their performance. The analysis shows the benefits of quantum solutions in different sectors of smart factories.

## 1. Introduction

**Figure 1:**
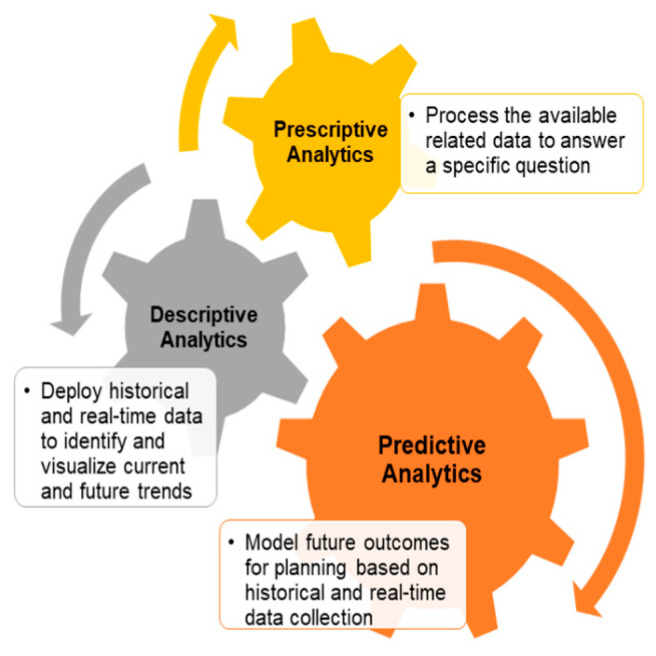
Smart factory-driven big data analytics categories.

History defines the term industrial revolution as the development from agriculture and a human-based economy to a machine-based industry. Starting with the deployment of steel into production, the discovery of new energy sources, such as coal, led to the innovation of machines and engines. The invention of machines was the major element that changed the production line by creating the concept of factories. Then, transportation and communication systems evolved, leading to an evolution in mass production and, finally, the stage of automation of the manufacturing process that radically changed the world we live in [1]. Moreover, with the fast development of communication technology, the concept of the industrial Internet of things (IIoT) became more integrated into our daily lives, and normal cities became smart cities with interconnected devices, sensors, machines, and systems. IIoT devices generate enormous amounts of data in different sectors, which created a new paradigm known as the big-data-based intelligence and analytics system that studies the impact of big data on smart factory organization [2]. Large companies are relying primarily on big data analysis for control, optimization, design, supply, and discovery. The results are mainly organized and visualized for more effective decision making. Smart factory-driven big data analytics can be divided into three main categories, as shown in Figure 1.

It is believed that big data is the fuel for companies and smart factories to improve production, reduce the cost of manufacturing, and manage risks, which explains why large companies are spending millions of dollars on big data analysis and hiring experts in databases, econometrics, visualization, statistics, and machine learning.

Data is important for smart factories; however, too much data can be challenging. In today’s society, and with the fast and continuous development of the IoT and network generations, the IIoT is facing a problem of raw enormous and heterogeneous data collected from different sectors. The large amount of collected data is getting harder and more complicated to classify and analyze, which is slowing down the processing phase. Furthermore, the security and privacy issues regarding data collection and communication are critical for an effective analysis. The data can be easily tampered with, manipulated, corrupted, or collected from the wrong nodes, which directly affects the analyzed results, leading to inaccurate decision making and costing smart factories huge losses. Moreover, the current computer power we are relying on is approaching its limits already. Based on Moore’s law, the number of transistors in each square inch on a microchip is doubling every year, which means that today’s transistors are reaching their end. Thus, it is inevitable to discover new forms of transistors and novel solutions for data processing [3].

Another issue with big data analysis for smart factories is digital carbon footprint; the information and communications technology (ICT) sector is reported to be contributing by 1.4 percent of the overall global carbon emission from unused switched-on base stations to complex and energy harvesting computations and processing at cloud servers, digital technologies are powerful tools that can be used either for better or for worse, depending on societal framing.

On the other hand, quantum computers and quantum information science (QIS) are viewed as the next area and solution for computation and data processing [4]. Quantum computers rely on the superposition of photons, which is a quantum mechanical phenomenon, and states that the polarization of each photon cannot be known unless it is measured, which means that the state of a qubit (the version of bits used in quantum computers) is both 0 and 1 at the same time. Another property of quantum computers is the entanglement of qubits, where the probability state of each qubit is directly affected by another entangled qubit. Saying two qubits are entangled, the moment we measure the state of one of them, we can immediately know the state of the other qubit even if they are polar apart. This property mathematically guarantees an exponential growth of qubit state probabilities with every qubit entangled in this system; given two entangled qubits, the probability of their state during measurement is four (00,01,10,11), and adding only one qubit to the system will double the probability range to eight, and so on. Quantum computers are promising an exponential growth of processing power that goes far beyond any supercomputer we have today. Google announced that their 53-qubit quantum chip managed to solve in a few seconds complex mathematical problems that took classical computers years to solve.

Quantum computing and quantum machine learning offer the potential to enhance data processing efficiency in various ways. Firstly, quantum computers can perform certain computations much faster than classical computers by using quantum bits (qubits) that can represent multiple states simultaneously, allowing for parallel processing and faster calculations. Secondly, quantum algorithms can optimize problems involving large amounts of data by efficiently searching through them and finding the best solutions. Thirdly, quantum computers can simulate complex physical systems that are hard to model using classical computers, which have applications in fields such as chemistry and materials science. Fourthly, quantum machine learning algorithms can help process and analyze unstructured data, which is difficult to process using traditional algorithms. Lastly, quantum computing has the potential to enhance data security by allowing for the creation of unbreakable encryption methods, which are currently challenging to implement. Overall, quantum computing and quantum machine learning have the potential to transform data processing and improve computational efficiency for a wide range of problems.

The future of smart factory analysis requires high processing power with low carbon emissions to create the goal of sustainable development. To this end, in this paper, we propose the deployment of quantum information technology in IIoT for optimized decision making, faster and effective data processing, reduced computation time which leads to an adequate reduction in carbon emission, and to maintain a scalable development environment in future smart factories.

The rest of our proposed research paper is organized as follows; Section 2 discusses the related state of the art regarding quantum solutions for smart factory and present our main key considerations followed in our system. Section 3 depicts the overview of our system model and explains possible applications of quantum algorithms in several industries. To prove our designed model, we study two case scenarios in Section 4 and analyze their performance. Section 5 provides the open research challenges. Finally, we conclude this paper with Section 6.

## 2. Related Work

To the best of our knowledge, and until the time of drafting this paper, there is no comprehensive literature review on possible applications of quantum algorithms in smart factories and industries. Some research discusses and analyzes a giving algorithm on one industry sector, yet there is no universal design that can be deployed for every industry and smart factory case. In this section, we review the existing research and related papers, analyze their contributions, and depict our key considerations.

### 2.1. Existing Studies

Luckow et al. [5] discussed in their research paper various possibilities of quantum implementations for the automotive industry. The authors investigated several issues within the industry that are, until today, considered complex problems to classical computers, such as robotic path optimization, vehicle configurations, system verification, route optimization in logistics, placement and distribution problems, strategic planning, tactical planning, operational planning, portfolio optimization, nanoscale functional materials development, engineering, design, and computer vision. As a solution, the authors depicted several quantum algorithms, including a quantum approximate optimization algorithm, a quantum adiabatic algorithm, Grover’s adoptive search algorithm, differential quantum circuits, variational quantum classifiers, and quantum neural networks. On the other hand, EL Azzaoui et al. [4] proposed the deployment of a quantum cloud system to solve the processing complexity of medical data. Their proposal also includes the usage of delegated cloud for secure and private data computation where the input, computation, and output are blindly performed. The paper solves the issue of molecule simulation and drug discovery processes in the healthcare industry. Yarkoni et al. [6] studied the possibilities of implementing quantum annealing (QA) processors for optimization problems across the industry. QA is a small processor developed by multiple quantum companies, notably D-Wave, and it allows the user to run quantum-based optimization algorithms for different problems. Based on the authors, QA can be implemented in mobility, scheduling and logistics, simulation, and finance, including portfolio optimization. Moreover, an IBM research team published a paper on the prospects of quantum computing applied to the finance industry. In this paper, Egger et al. [7] depicted the applicability and potential of quantum computers for several problems in the finance sector, including banking, the financial market, and insurance. According to the research team, quantum computers shall be beneficial in customer identification, financial products, monitoring transactions, and customer retention using a quantum AE algorithm. Quantum AE estimates parameters with a convergence rate of $O(\frac{1}{M})$, where *M* refers to the number of quantum samples. This algorithm quadratically speeds up the processing phase compared to classical algorithms.

These state-of-the-art papers presented the utilities of quantum computers and algorithms as effective solutions for industries and smart factories. However, until the time of writing this paper, there is no paper or research work that discusses the benefits of quantum implementation in smart factories at large with different industries. Moreover, these papers do not include a practical case study or scenario to visualize the results of using quantum computers. Thus, in this paper, we depict the benefits and applications of quantum computers to various smart factories and industries. Furthermore, we study some real-world case scenarios where we implement quantum algorithms, and we analyze the obtained results. Interventional studies involving animals or humans, and other studies that require ethical approval, must list the authority that provided approval and the corresponding ethical approval code. Table 1 summarizes the discussed research works.

### 2.2. Key Considerations

The primary considerations of the proposed solutions are depicted as follows:

- **Scalability**: Smart factories and industries, in general, rely on big data collected from different sectors. The heterogeneity of the data contributes to the complexity of the system. The more data there is to be processed, the more time it will take to classify and the more complex the system will be. Thus, creating a scalable computational system for smart factories and industries to use is required to maintain the fast-growing needs of smart factories, and quantum algorithms can surely ensure that.
- **Efficiency**: The efficiency of the big data processing phase is another condemnatory demand for developed smart factories. Yet, the large amount of data facing smart factories is not allowing the system to be efficient enough. Smart factories should be able to classify the acquired data efficiently in order to process it and extract the required information for smart factory development. The current solutions using classical computers and algorithms are struggling to manage these data, which leads to more processing time and less effective results. To this end, we believe that quantum computers and quantum-based algorithms can be a suitable solution for today and future smart factories.
- **Integrity**: Currently, data breaches are the most critical problem facing the security of smart factories around the world. Mostly, data theft and breaches happen during the processing phase at the server level, where smart factory-related data, such as product information or personalized customer information, are stored [8,9]. Moreover, data manipulation can cause severe losses to smart factories. Surprisingly, even tech giants such as Facebook have been through this issue, and it costs them millions of dollars to gain the trust of their clients back. In the case of the finance industry, such as banks, data theft and breaches can be critical as they can lead to the exposure of sensitive information, such as monetary transactions and credit card information, to the public. Using quantum-based cryptography, we can enhance the security of smart factories and ensure the privacy and security of their data during the processing phase.
- **Availability**: Smart factories should be able to access their data and results anywhere and at any time; ensuring the availability of information and data is critical to various smart factories and industries. To this end, securing smart factory servers from cyberattacks such as DDoS is essential [10,11].

## 3. Proposed Quantum Approach for Secure Decision Making in IIoT-Based Smart Factory System

**Figure 2:**
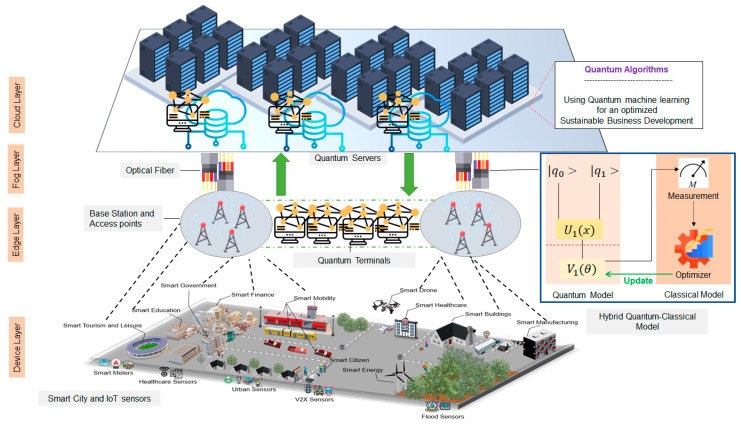
Quantum-based smart factory solutions system overview.

In this paper, we first discuss possible applications of quantum computers and quantum algorithms for several IIoT system models and industries. Moreover, we propose two real-world case studies: the first one is logistic system optimization using a quantum approximate optimization algorithm (QAOA) that can be applied in almost all smart factories that deal with logistics and product shipping, and the second is a quantum neural network (QNN) implementation for finance sector where the algorithm can be used for fast, scalable, and more efficient smart factory forecasts, price-related data analysis, and future opportunity discovery. The proposed system is universal and can be deployed by various industries for their smart factory modeling and optimization using quantum algorithms. The system can be divided into four main layers, notably the device layer, edge layer, fog layer, and cloud layer, as depicted in Figure 2.

### 3.1. General System Overview

The proposed system is flexible and designed to be able to run different quantum algorithms based on the needs of each smart factory and industry:

- **Device layer**: The first layer of our proposed system is the device layer; The first layer of our proposed system is the device layer; it holds different IoT devices and data sources that smart factories are using to collect data based on their needs. These data include personal information and customers, such as name, gender, IP address, and identification number. The second type of collected data is engagement and behavioral data. These data help smart factories to understand their clients and personalize unique products and commercials for them, which is regarded as the most essential information that almost every smart factory relies on in the era of social platforms. Another type of data that can be collected is regarding the smart factory itself, such as data related to production collected from manufacturers or related to products, logistics, and shipping.
- **Edge Layer**: The second layer in the proposed system is the edge layer. In this layer, we implement quantum terminals, which are small quantum processors with a minimum of one qubit size that are capable of converting classical bits representing data obtained from the device layer into qubits that are understandable by the quantum cloud server at the cloud layer. This method eliminates the need for quantum programmers or quantum specialists and automatically performs the required quantum computations at the cloud layer for optimized and efficient smart factory decision making. The deployed method at this layer has been proved previously in our published paper [4].
- **Edge Layer**: At the edge layer, we implement a hybrid quantum–classical model, which is capable of performing hybrid computations; that is, if a certain operation does not require quantum processing, it can immediately perform using classical machine learning methods, which contributes to a much faster and more scalable system. Moreover, the proposed model can be used in quantum machine learning in order to optimize performance. More details about this model are discussed below when we discuss the case study of QNN.
- **Cloud Layer**: The final layer in the proposal is the cloud layer where the quantum server resides. The quantum server at the cloud layer is powered by quantum machines to perform all sorts of requested computations from the smart factory client. The requested analyses are executed exponentially faster, are scalable, and are more efficient than any classical computer.

### 3.2. Quantum Application for Smart Factory Development

In this section, we discuss various implementations of quantum algorithms and quantum solutions that can be applied to smart factories in order to enhance and optimize their analysis. For instance, quantum optimization algorithms based on gradient descent [12,13,14,15], such as QAOA, can be used in supply chains, logistics optimization, transportation routing, process planning, pricing and promotion optimization, products portfolio optimization, fabrication and production optimization, energy distribution optimization, financial modeling, credit organization, insurance optimization, emergency planning, protein folding prediction and drug discovery in the medical sector, and network optimization [16,17,18,19,20]. Quantum machine learning, such as QNN, can be deployed as well in various sectors such as material discovery, material research, finance analytics, precision medicine therapies, and drug structure [21,22,23]. Quantum search algorithms, such as Grover’s algorithm, can be used in complex and unstructured databases, which can be very efficient in smart factory modeling and IIoT analytics. Quantum-based security algorithms such as quantum key distribution, quantum one-time pad, and quantum random number generation can be useful to secure smart factory and their data from cyberattacks and can be deployed for fraud detection, anomaly analysis, intrusion detection, cyber-risk management in smart factory, and product risk analysis [24].

Smart factories and industries can profit from quantum applications and algorithms to secure their data and optimize their processing, analysis, and computation. The future of smart factories relies on futuristic technologies; thus, we believe that smart factories and industries should start deploying quantum solutions into their products in order to adapt to the fast development of the quantum era.

## 4. Case Study and Evaluation

In this section, we further discuss our proposed system using two real-world case studies. The first one is based on a quantum neural network for the finance sector, where we deploy QNN for forecasting, analyzing, and discovering future smart factory opportunities. The scenario we studied is based on the designed system explained above. The second case study is regarding the max-cut problem for logistic systems in smart factories, which is solved using QAOA.

### 4.1. QNN-Based Financial Forecast in Smart Factory

In the case of finance and IIoT, the input values are raw data continuously collected from the device layer, such as sale numbers, losses, product prices, and so on. Using the quantum terminal, we can generate the rotation angle for the quantum rotation gate (QRG). The output of variational encoding is used as an input for the QNN algorithm. QNN classifies the data, labels it, and processes it; the output is a real-time analysis result that can be immediately visualized to understand the future direction of a given market. The trained model is then sent to the cloud layer. In this phase, future prediction on the market and pre-planned smart factory model is computed. Note that at this level, only the trained model is sent as an input and not the data, which increases the security and privacy of the smart factory as the data is blindly computed, and it reduces the burden on the native AI-based edge layer since the upcoming possible smart factory models are pre-calculated. To evaluate the proposed solution, we have used IBM Quantum Lab’s Qiskit Software version 0.19.0 and Python version 3.8.10 under a Linux operation system with four CPUs and 7.68 (Gb) memory usage. We consider that the data is continuously fed to the native intelligent edge layer where the QNN algorithm runs. First, the raw data is collected from the devices to their respective base station where the quantum terminal is implemented. The quantum machine terminal (QMT) serves as an intermediary between classical devices and quantum servers, enabling the compilation of classical bits into qubits that are readable by the quantum server and vice versa. With at least one qubit, the QMT is a compact quantum machine.

The variational encoding converts the classical data bits into a quantum state. The QNN algorithm has to classify the data; thus, at this point, there are two different techniques to use. The first technique involves classification with a circuit QNN, which is used to classify within a neural network classifier and return the d-dimensional probability vector as an output (d is the number of outputs), thus resulting in a probability distribution. The other method that can be used is the variational quantum classifier (VQC). The VQC uses extensions to multiple classes to map from the bitstring to the classification, resulting in a probability vector.

**Figure 3:**
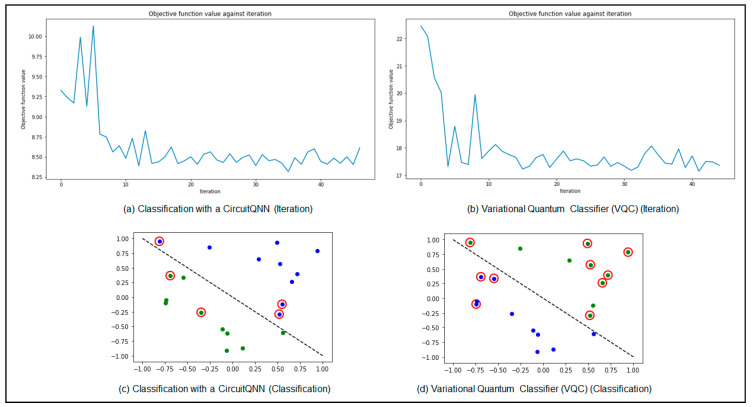
Results comparison between CircuitQNN and VQC (iteration and classification): (**a**) classification with a CircuitQNN (iteration); (**b**) variational quantum classifier (VQC) (iteration); (**c**) classification with a CircuitQNN (classification); (**d**) variational quantum classifier (VQC) (classification).

**Figure 4:**
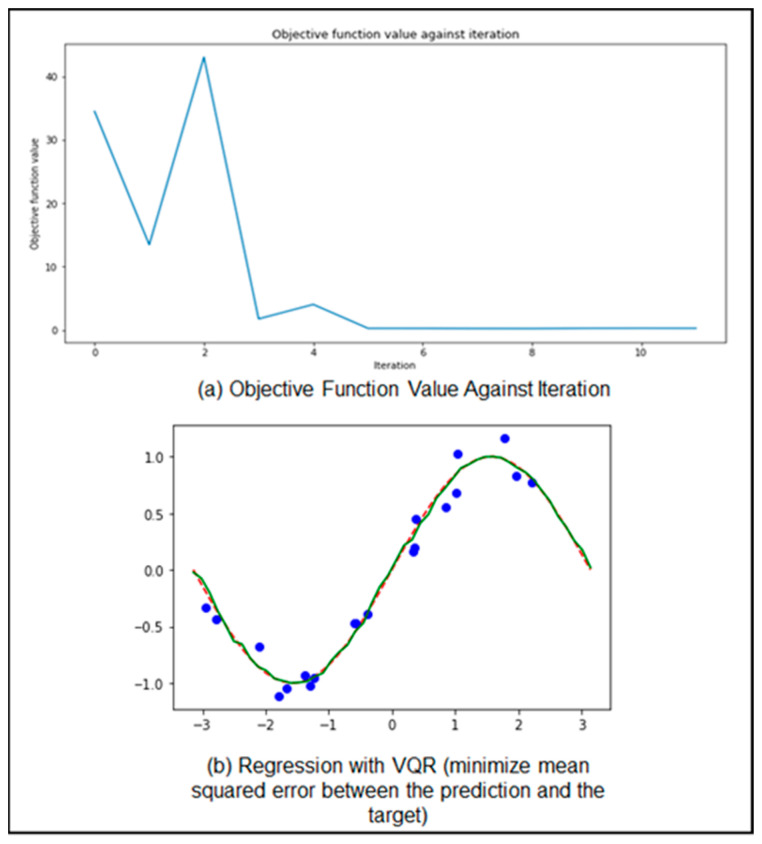
VQR regression output. (**a**) Objective function value against iteration; (**b**) regression with VQR (minimize mean squared error between the prediction and the target).

VQC is one of the best solutions for quantum neural network (QNN) classification problems as it is noise-resistant and scores a high success rate in machine learning (ML) models [10,11]. VQC deploys a classical optimizer model as depicted that continuously updates the parameters back to the QNN model, reducing the cost, time, and iterations, thus making it a hybrid (quantum–classical) algorithm. Figure 3 shows the output of running both circuit QNN and VQC; the results prove the outstanding performance of the hybrid VQC technique compared to the circuit QNN technique. For the regression, we used a variational quantum regressor (VQR) which is similar to VQC. Figure 4 shows the performance of VQR using the L2Loss function for lesser iterations. The graph depicts a very minimized mean squared error between the prediction and the target.

### 4.2. Quantum Machine Learning for Optimized Route Selection in Smart Logistics

In this case study, we present a feasible solution for implementing quantum machine learning for a scalable and efficient smart factory financial prediction. Our proposed system uses a quantum terminal, which is a one-qubit processor that is capable of encoding classic bits from the collected data into quantum states. Moreover, we used a hybrid QNN model where the classical layer is used to optimize the output and update the quantum model to reduce the cost of computation and execution. The results prove the feasibility of the proposed model as it is capable of providing a real-time forecast based on the provided data. On top of that, we propose that the trained model using QNN should be sent to the cloud layer for further computations to predict future finance results, such as share prices, thus reducing the future complexity on the intelligent edge as the prediction reached 96%. This method can be used as the first step for future research directions on using small quantum processors at the edge to improve the smart factory’s scalability and the quality of experience, reduce the cost, and create a total self-learning optimized architecture.

Smart factories around the world rely on their logistic system either for product delivery, raw material storage, or transportation. The main problem facing smart factories is reducing the shipping cost by optimizing the route and minimizing the number of nodes that a delivery person needs to stop at, which is well-known as the traveling salesman problem. This problem is considered an NP-complete problem where its representative complexity exponentially increases with the number of nodes. To solve this problem, we deploy QAOA, where the data is collected from the device layer; in this case, the collected data represent the GPS location and the address that it should stop by them. We consider some conditions where the salesman should start and end at the same location (headquarter of the smart factory, for example), the salesman cannot disappear from the system, and they should visit each and every node only once. This state can be represented using the Hamiltonian problem depicted in the following equation:

(1) $$H_{p}=\frac{1}{2}\left(Z_{0}⊗Z_{1}⊗I_{2}⊗I_{3}\right)+\frac{1}{2}\left(I_{0}⊗Z_{1}⊗Z_{2}⊗I_{3}\right)+\frac{1}{2}\left(Z_{0}⊗I_{1}⊗I_{2}⊗Z_{3}\right)+\frac{1}{2}\left(I_{0}⊗I_{1}⊗Z_{2}⊗Z_{3}\right)$$

The mixer Hamiltonian $H_{B}$ is represented in (2).

(2) $$H_{B}=\left(X_{0}⊗I_{1}⊗I_{2}⊗I_{3}\right)+\left(I_{0}⊗X_{1}⊗I_{2}⊗I_{3}\right)+\left(I_{0}⊗I_{1}⊗X_{2}⊗I_{3}\right)+\left(I_{0}⊗I_{1}⊗I_{2}⊗X_{3}\right)$$

At this stage, the unitary gate *U* corresponding to $H_{B}$ and $H_{P}$ is depicted as follows, where the product is related to a rotation on each qubit.

(3) $$U\left(H_{B}\right)=e^{-i\beta H_{B}}=e^{-i{\beta X}_{0}}e^{-i{\beta X}_{1}}e^{-i{\beta X}_{2}}e^{-i{\beta X}_{3}}$$

(4) $$U\left(H_{P}\right)=e^{-i\gamma H_{P}}=e^{-i\gamma Z_{0}Z_{1}}e^{-i\gamma Z_{1}Z_{2}}e^{-i\gamma Z_{2}Z_{3}}e^{-i\gamma Z_{0}Z_{3}}$$

In order to start the QAOA, we must initialize the quantum states using unitary gate $U\left(H_{P}\right)=e^{-i\gamma H_{P}}$ and operations such as deploying the mixing unitary $U\left(H_{B}\right)=e^{-i\beta H_{B}}$. Finally, we measure the Z-basis gate in order to obtain the optimal route selection for the salesman as follows:

(5) $$<\psi \left(\beta_{opt},\gamma_{opt}\right)\left|H_{P}\right|\psi \left(\beta_{opt},\gamma_{opt}\right)>$$

**Figure 5:**
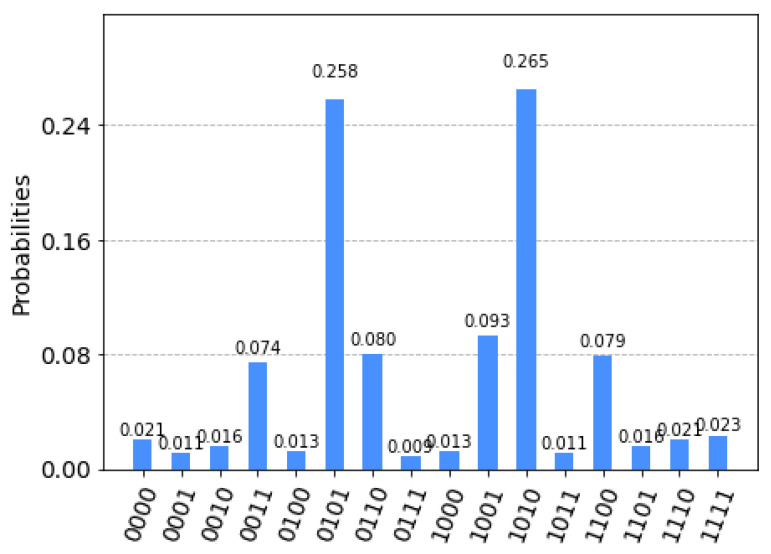
Optimal route selection probability.

Figure 5 depicts the probability of finding the optimal route results obtained by deploying this method on IBM Quantum Cloud. The results show a high probability at the 0101 and 1010 qubits of 0.258. Based on the highest probability, the most optimal route shall be selected. The results obtained are depicted in Table 2.

## 5. Open Research Challenges

The implementation of quantum information science into smart factories and IIoT will indeed exponentially speed up the development of smart factories by increasing the processing time and efficacy [25]. However, quantum solutions are currently in the earliest implementation stages, which lead to various challenges that urge to be addressed. These challenges include quantum error correction caused by various factors, such as noise and decoherence. Developing efficient quantum error correction algorithms is crucial to mitigate these errors and ensure the reliability of quantum solutions in smart factories. Another challenge is quantum sensors, where developing efficient and reliable sensors can be challenging. Moreover, quantum solutions for smart factories may require the development of a new quantum network infrastructure to enable communication between quantum devices. Developing efficient quantum network infrastructure that can scale to large numbers of devices is an open research challenge. These open research challenges require significant investment in research and development to build the necessary skills and expertise to enable the deployment of quantum solutions in smart factories. However, we believe that our proposed solution using a hybrid approach rather than a direct quantum approach is the most feasible solution as it requires less quantum hardware. Moreover, using the quantum terminal represented by the quantum one-qubit chip reduces the need to have a quantum computer at the device layer, which reduces the cost of implementation and makes the proposed solution much more sustainable, efficient, and possible.

Quantum machine learning (QML) has the potential to revolutionize certain areas of machine learning, but there are currently several limitations that need to be addressed before it can be widely adopted. Some of these limitations include the current limitations of quantum hardware, the limited availability of quantum resources, the lack of standardization of QML algorithms, the challenges associated with accessing large amounts of data, and the shortage of experts with the skills and knowledge required for QML. Despite these challenges, researchers and industry experts are actively working to overcome these limitations, and it is expected that QML will continue to advance and become more widely adopted in the near future.

## 6. Conclusions

The fast development of the quantum era is growing exponentially, with companies such as D-Wave announcing their quantum Annealing into the market that allows companies to run quantum algorithms for their needs. Moreover, the large and heterogenous data collected from various IoT devices is increasing the complexity of computation for smart factories. Industries are developing at a fast rate around the world, with technologies such as machine learning and AI being enhanced; however, the complexity of computation is standing against smart factory development. To this end, in this paper, we discuss the implementation of quantum solutions for sustainable smart factory development. We depict various applications where quantum algorithms could improve smart factory scalability and productivity. Moreover, we design a universal system model where smart factories would not need to acquire quantum computers to run quantum algorithms based on their needs; instead, they can use quantum cloud servers and quantum terminals implemented at the edge layer to help them run the desired quantum algorithms without the need of an expert. To prove the feasibility of our model, we implement two real-world case studies and evaluate their performance. The analysis shows the benefits of quantum solutions in different smart factories. We hope this paper will be a stepping stone for further research and application of quantum solutions for smart factories around the world. The results show promising performance for quantum machine learning approaches to optimize and enhance the efficiency of IIoT systems.

## Figures and Tables

**Table 1:** Comparison of related works.

Paper	Year	Scalability	Efficiency	Integrity	Availability
EL Azzaoui et al. [4]	2022	X	O	O	O
Luckow et al. [5]	2021	O	O	X	X
Yarkoni et al. [6]	2022	O	X	X	O
Egger et al. [7]	2020	O	O	X	X
Our contribution	2023	O	O	O	O

**Table 2:** Performance of quantum machine learning for smart logistics.

Task	Performance
Route Optimization Computing Latency	8.6 (s)
Route Optimization Decision Latency	5 (ms)
Successful Optimized Route Probability	91%
System Scalability Improvement	23%

## Data Availability

Data is contained within the article.

## Abbreviations

Symbols	Description
QIS	Quantum Information System
QA	Quantum Annealing
AE	Amplitude Estimation
QAE	Quantum Amplitude Estimation
*M*	Number of Quantum samples
QAOA	Quantum Approximate Optimization Algorithm
QNN	Quantum Neural Network
QRG	Quantum Rotation Gate
VQC	Variational Quantum Classifier
QMT	Quantum Machine Terminal
$H_{p}$	Hamiltonian problem
$H_{B}$	Mixer Hamiltonian
U	Unitary Gate
Z	Pauli-Z Gate
I	Identity Matrix
X	Pauli-X Gate
